# Ca^2+^-mediated Mitochondrial Reactive Oxygen Species Metabolism Augments Wnt/β-Catenin Pathway Activation to Facilitate Cell Differentiation*

**DOI:** 10.1074/jbc.M114.573519

**Published:** 2014-08-14

**Authors:** Tareck Rharass, Heiko Lemcke, Margareta Lantow, Sergei A. Kuznetsov, Dieter G. Weiss, Daniela Panáková

**Affiliations:** From ‡ Electrochemical Signaling in Development and Disease, Max-Delbrück-Center for Molecular Medicine, Robert-Rössle-Strasse 10, D-13125 Berlin-Buch and; § Cell Biology and Biosystems Technology, Institute of Biological Sciences, and; ¶ Live Cell Imaging Center, University of Rostock, Albert-Einstein-Strasse 3, D-18059 Rostock, Germany

**Keywords:** Calcium Signaling, Mitochondrial Metabolism, Reactive Oxygen Species (ROS), Redox Signaling, Wnt Pathway, Dishevelled, Human Neural Progenitor Cells, Nucleoredoxin

## Abstract

Emerging evidence suggests that reactive oxygen species (ROS) can stimulate the Wnt/β-catenin pathway in a number of cellular processes. However, potential sources of endogenous ROS have not been thoroughly explored. Here, we show that growth factor depletion in human neural progenitor cells induces ROS production in mitochondria. Elevated ROS levels augment activation of Wnt/β-catenin signaling that regulates neural differentiation. We find that growth factor depletion stimulates the release of Ca^2+^ from the endoplasmic reticulum stores. Ca^2+^ subsequently accumulates in the mitochondria and triggers ROS production. The inhibition of mitochondrial Ca^2+^ uptake with simultaneous growth factor depletion prevents the rise in ROS metabolism. Moreover, low ROS levels block the dissociation of the Wnt effector Dishevelled from nucleoredoxin. Attenuation of the response amplitudes of pathway effectors delays the onset of the Wnt/β-catenin pathway activation and results in markedly impaired neuronal differentiation. Our findings reveal Ca^2+^-mediated ROS metabolic cues that fine-tune the efficiency of cell differentiation by modulating the extent of the Wnt/β-catenin signaling output.

## Teaser

**Background:** Dissociation of the Wnt/β-catenin pathway effector Dishevelled from its complex with nucleoredoxin is a redox-sensitive process, yet the ROS sources remain elusive.

**Results:** Mitochondrial Ca^2+^ influx stimulates endogenous ROS production and mediates Wnt/β-catenin pathway activity.

**Conclusion:** Ca^2+^-mediated ROS production modulates the signaling efficiency of the Wnt/β-catenin pathway.

**Significance:** Metabolic states influence fundamental and developmental signaling to drive cell differentiation.

## Introduction

Excessive production of reactive oxygen species (ROS)^2^ leads to oxidative stress and cell damage in a number of pathologies (1). Growing evidence suggests that at low concentrations ROS act as second messengers in a variety of cellular processes and drive redox-dependent events (2). Recently, ROS production has been implicated in the regulation of such signaling pathways as ERK1/2 and JNK1/2 during directional cell migration, Akt in cell proliferation, and Wnt/β-catenin in proliferation, regeneration, and embryonic patterning (3–8).

The involvement of ROS in regulating Wnt signaling was first proposed by Funato *et al.* (9, 10). They reported that Dishevelled (DVL) is kept inactive in the cytoplasm by forming a complex with nucleoredoxin (NRX), a ubiquitously expressed member of the thioredoxin antioxidant superfamily. DVL has so far been identified as an intermediate in all known aspects of Wnt signaling, and DVL translocation from the cytoplasm to the plasma membrane is the critical step in the activation of the Wnt signal transduction (11). Funato *et al.* (9, 10) showed that upon treatment of cells with an exogenous pro-oxidant compound, DVL was released from its complex with NRX, which leads to the stimulation of the Wnt/β-catenin pathway. The data suggested that the changes in intracellular ROS levels might positively regulate the Wnt/β-catenin pathway by modulating DVL availability to transduce the Wnt signal. One source of physiologic ROS can be attributed to the elevated enzymatic activity of plasma membrane NADPH oxidases (5, 6). However, the role of the major cellular ROS source, mitochondrial ROS, in the activation of Wnt/β-catenin signal transduction remains incompletely understood.

**FIGURE 1.**
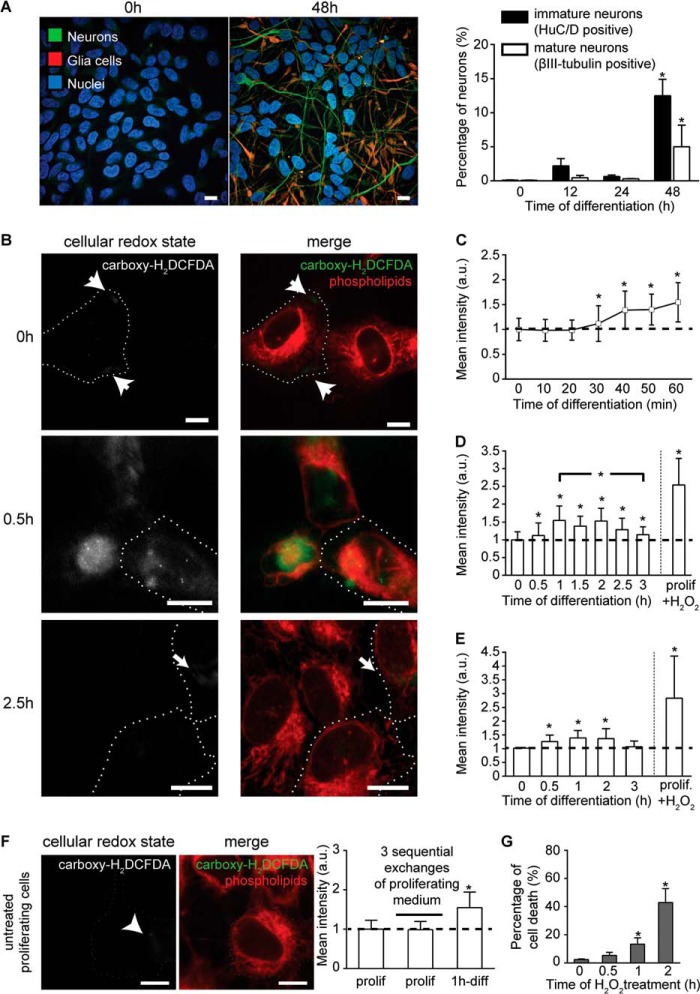
**Induction of differentiation alters intracellular redox balance.** *A,* confocal images of neurons (βIII-tubulin, *green*) and glia cells (*red*) at 0 h (proliferating cells) and 2 days after initiation of differentiation. Nuclei are shown in *blue*. Mature neurons were quantified based on βIII-tubulin labeling. Immature neurons were quantified through the labeling of the neuronal RNA-binding protein Hu-antigens C and D (*HuC/D*) that is expressed earlier than βIII-tubulin. *n* = ∼9000 cells per time point. *B,* confocal images of redox state (grayscale; *green* in merge) at 0, 0.5, and 2.5 h of differentiation. *White arrows* indicate faint signal at 0 and 2.5 h. Phospholipids (*red*) and cell boundaries (*dotted white lines*) are shown. *C,* kinetics of the cellular redox state measured as mean fluorescent intensity at 10-min intervals over the 1st h of differentiation. Significant increase appears at 30 min of differentiation. *n* = ∼150 cells per time point. *D,* kinetics of the cellular redox state measured as mean fluorescent intensity at 0.5-h intervals over the first 3 h of differentiation. Redox state reaches baseline levels after 3 h. *n* = ∼150 cells per time point. *E,* kinetics of the cellular redox state measured as mean fluorescent intensity at 0.5-h intervals over the first 3 h of differentiation using flow cytometry. *a.u.*, arbitrary units. *F,* confocal images of intracellular redox state (grayscale; *green* in merge) after three sequential exchanges of proliferating medium in pre-stained proliferating cells. Phospholipids are in *red*. Mean fluorescent intensities show change in redox state only in differentiating cells. *n* = ∼50 cells per time point. *G,* cytotoxic effect of 3 mm H_2_O_2_ assessed with MTT. *, *p* ≤ 0.05. *Error bars*, S.D. *Scale,* 10 μm.

Upon withdrawal of epidermal and basic fibroblast growth factors (EGF and bFGF), immortalized human neural progenitor ReNcell VM197 cells (hereafter hNPCs) differentiate within 3 days into neurons and glial cells (Fig. 1*A*) (12, 13). Expression analysis of a number of WNT ligands, their relative receptors, and Wnt target genes showed that Wnt/β-catenin signaling regulates the neuronal cell fate commitment of hNPCs within 24 h of differentiation (14, 15). A detailed spatiotemporal profile of total and phosphorylated protein levels of key components of the Wnt/β-catenin transduction pathway LRP6, DVL2 (dishevelled segment polarity protein 2), and β-catenin revealed their activation already within the first 4 h of differentiation (15). The tightly regulated temporal component of the Wnt/β-catenin pathway activation in hNPCs prompted us to investigate potential changes in mitochondrial ROS metabolism immediately after growth factor (GF) depletion. We sought to explore whether ROS levels increase prior to and whether they are required for Wnt/β-catenin pathway activation. Moreover, we sought to find what mechanisms might be triggering ROS metabolism.

Here, we provide evidence that in hNPCs, endogenous mitochondrial ROS production is markedly increased as a result of GF depletion at the onset of neural differentiation and that ROS production precedes the activation of the Wnt/β-catenin pathway. We find that GF depletion stimulates the release of Ca^2+^ from endoplasmic reticulum stores through the inositol 1,4,5-triphosphate receptor, type 1 (ITPR1). Subsequently, a fraction of Ca^2+^ flows into the mitochondria via the mitochondrial calcium uniporter (MCU). This increase in mitochondrial Ca^2+^ is required for elevated ROS production. The inhibition of Ca^2+^ efflux via ITPR1 or Ca^2+^ influx via MCU attenuates the ROS metabolism and prevents the dissociation of DVL2 from its inactive pool sequestered by NRX in the cytoplasm. Moreover, the robust activation of DVL2 is blocked as we observe a significant decrease in the β-catenin nuclear accumulation, attenuated expression of Wnt/β-catenin signaling target genes, and impeded neuronal differentiation. Our data reveal that Ca^2+^-mediated mitochondrial ROS metabolism is directly involved in the regulation of early events of Wnt/β-catenin transduction and imply that the cellular metabolic state has an integral role in the Wnt/β-catenin pathway.

## EXPERIMENTAL PROCEDURES

### 

#### 

##### Cell Culture and Treatment

The immortalized human neural progenitor cell line ReNcell VM197 (ReNeuron) was derived from the ventral midbrain of 10-week-old human fetal neural tissues. Cells proliferate in laminin (R&D Systems) pre-coated flasks under human bFGF (Invitrogen) and human EGF (Sigma) stimulation in proliferating medium (DMEM/F-12 medium with B27 neural cell supplement, l-glutamine, heparin, and gentamycin) (all Invitrogen) as described previously (13). The differentiation of subconfluent (70–80%) cell layers is induced by discarding the proliferating medium followed by Hanks' balanced salt solution (Invitrogen) rinsing and replacement with differentiating medium (*i.e.* medium without growth factors). Treatment of cells with 0.5 or 10 μm ruthenium red (RuR) (Sigma) was performed for 3 h as follows: 1 h of pretreatment with the reagent prior to the induction of differentiation, followed by a post-treatment up to the 2nd h of differentiation; to reverse the drug effect, the drug-containing differentiating medium was replaced by a drug-free medium after Hanks' balanced salt solution rinsing. Proliferating cells were also pretreated with lithium chloride (LiCl; 20 mm, 1 h) and *N*-acetyl-l-cysteine (NAC; 10 mm, 24 h) (both Sigma), followed by post-treatment up to 24 and 72 h after the induction of differentiation, respectively. Cytotoxicity was assessed by trypan blue exclusion assay (Invitrogen) or MTT test (Sigma). Cells seeded in Petri dishes were trypsinized and stained with trypan blue; the cell counting was performed in triplicate and each condition assessed in duplicate. For the MTT assay, cells were seeded in 96-well plates and incubated for 2 h with 50 μg/ml MTT prior to lysis; absorbance at 550 nm was measured with 8 wells per experimental conditions for each experiment. Data were obtained from three independent experiments.

##### Cell Transfection

Cells were transfected with following human siRNAs as per the manufacturer's instructions (GE Healthcare): SMARTpool Accell mitochondrial calcium uniporter (*MCU*), SMARTpool Accell inositol 1,4,5-triphosphate receptor, type 1 (*ITPR1*), and Accell nontargeting pool (negative control). Briefly, 1 μm siRNA was mixed to Accell siRNA delivery medium (GE Healthcare) supplemented with EGF, bFGF, and B27 neural cell supplement and incubated with cells for 72 h prior to the induction of differentiation.

##### Confocal Laser Scanning Microscopy Equipment and Settings

Fluorescent images of fixed cells were acquired with LSM 710 NLO (Zeiss) and TCS SP5 and TCS SP8 (Leica) confocal microscopy systems. Live cell imaging was performed using TCS SP2 (Leica) and A1 (Nikon) confocal microscopes. Live cell samples were observed in a 37 °C humidified incubation chamber supplied with a CO_2_ enrichment system. Cell shape and integrity were observed in transmitted light to ensure no cell death occurred during the experimental time frame. The parameter settings (detector gain and offset, pinhole size, laser power, confocal section, zoom factor, line and frame averaging) were kept constant for all comparative sets of experiments. With these settings, no photobleaching was detected after several repeated measurements on the same microscopic field. Brightness/contrast adjustments were applied equally to every pixel in the images (*i.e.* maximum projections) and for each comparative set (*e.g.* proliferation *versus* differentiation) using Fiji/ImageJ. Adjustments were performed on individual color channels before merging images. No change to γ settings was applied. Regions of interest were set individually, *i.e.* for each cell of the population in the images, based on cell boundaries to calculate the mean fluorescence intensities (ratio of the sum of fluorescence intensity emitted in the regions of interest to the amount of pixels in the regions of interest). For each image, the background fluorescence was subtracted, and values were normalized. All data were obtained from at least three independent experiments. For each time point or treatment, at least 10 images per experiment were recorded. Results are shown as means ± S.D. arbitrary units.

##### Redox Balance and ROS Levels

Live cell imaging of intracellular redox state and mitochondrial ROS metabolism were, respectively, performed with TCS SP2 and A1 confocal microscopes, using the redox indicator carboxy-H_2_DCFDA (10 μm, 1 h) (16) and MitoTracker red CMXRos (50 nm, 45 min) (17), respectively. Staining of phospholipids or microtubule network using Nile red (10 μm, 10 min) or tubulin Tracker^TM^ Green (100 nm, 0.5 h) (all Invitrogen) allowed discriminating the cell boundaries for quantification of the mean fluorescence intensities. Nuclei were stained 10 min with 2 μm Hoechst 32258 (Sigma). All stainings were done at 37 °C, 5% CO_2_, in the dark. Between each staining three washing steps were achieved with pre-warmed culture medium. For the short term kinetic experiments (*i.e.* 1 h time line), proliferating cells were pre-loaded with the appropriate dyes and then the differentiation was initiated by GF removal, and the mean intensities were measured every 10 min. For the long term measurements (*i.e.* up to 3 h), the dyes were loaded right after the differentiation was induced, and measurements were performed every 30 min.

##### Flow Cytometry

Flow cytometry was performed to determine intracellular redox state and ROS levels. Cells were stained with carboxy-H_2_DCFDA or dihydrorhodamine 123 (1 μm, 0.5 h) (Invitrogen), a nonspecific indicator for intracellular ROS (16). Staining was performed in Hanks' balanced salt solution complemented with 14 mm HEPES and 0.9% NaCl (all Carl Roth). Mean fluorescence intensities in a total of 10^4^ events (*i.e.* cells) were determined in each sample using EPICS XL-MCL flow cytometer system (Beckman Coulter). An unstained cell sample was carried along as a control for autofluorescence. Data were analyzed for four independent exposure experiments measured in duplicate. 1–3 mm hydrogen peroxide (H_2_O_2_) (Invitrogen) and 5 μm phorbol 12-myristate 13-acetate (Sigma) were used as positive controls.

##### Ca^2+^ Imaging

Endoplasmic reticulum (ER) and mitochondrial Ca^2+^ fluxes were monitored with A1 confocal microscope using mag-fluo-4 AM (5 μm, 0.5 h) (18) and x-rhod-1 AM (2 μm, 0.5 h) (19), respectively. Fura red AM (20 μm, 0.5 h) (all Invitrogen) was used to assess cytosolic Ca^2+^ levels (20, 21) with a TCS SP2 confocal microscope. 1–10 mm caffeine (CAF) (Calbiochem) was used as positive control. Fura red, although presented as a ratiometric Ca^2+^ indicator through a dual excitation wavelength mode (440 and 488 nm for Ca^2+^-bound and -unbound forms, respectively), is rarely used this way because of the weak quantum yield of the Ca^2+^-bound form (21). Also, fura red was used here as a single wavelength emission dye, *i.e.* the Ca^2+^-unbound form. An increase of cyto-Ca^2+^ levels leads to a decrease of the measured fura red intensity due to the binding of Ca^2+^ (*i.e.* reduction of the pool of Ca^2+^-unbound form of the dye). Results were presented as *F*_0_/*F* ratio (*F*_0_ and *F* corresponding to the mean fluorescence intensities measured within proliferating cells and differentiating or treated cells, respectively).

##### Western Blotting

Protein extraction from the cells was achieved using Triton lysis buffer supplemented with protease and phosphatase inhibitor mixtures (Sigma). Cell lysates were loaded with Laemmli buffer, centrifuged for 10 min, and boiled for 5 min. Proteins were separated by SDS-PAGE using 10% SDS-polyacrylamide gel and transferred in a wet condition onto a nitrocellulose membrane (Amersham Biosciences). After blocking with 5% nonfat dry milk, proteins were labeled overnight with primary antibodies as follows: goat anti-NRX (R&D Systems, AF5719; dilution 1:2000); rabbit anti-DVL2 (Cell Signaling, 3216; dilution 1:500); and rabbit anti-GAPDH (Santa Cruz Biotechnology, sc-25778; dilution 1:800) used as stable housekeeping protein. Proteins were probed for 1 h with HRP-conjugated secondary antibodies as follows: anti-goat (Santa Cruz Biotechnology, sc-2020; dilution 1:50,000) and anti-rabbit (Sigma, A9169; dilution 1:80,000). Signals were detected with ECL Western blot detection reagent (GE Healthcare) and quantified with Fiji/ImageJ. Relative signal intensities were normalized to GAPDH.

##### Immunocytochemistry

Cells grown on glass coverslips precoated with poly-d-lysine and then with laminin were fixed 20 min with 4% paraformaldehyde and 4% sucrose in PBS followed by 10 min of quenching with 50 mm NH_4_Cl and 5 min of permeabilization with 0.2% Triton X-100. Nonspecific binding sites were blocked for 1 h with 1% gelatin (all Sigma). Cells were loaded 1 h with primary antibodies as follows: rabbit anti-DVL2 (Santa Cruz Biotechnology, sc-13974; dilution 1:200); goat anti-NRX (Santa Cruz Biotechnology, sc-161973; dilution 1:200); mouse anti-α-tubulin (Sigma, T5168; dilution 1:1000); mouse anti-β-catenin-Alexa488-conjugate (Pharmingen, 562505; dilution 1:200); mouse anti-glial fibrillary acidic protein-Cy3-conjugate (Sigma, C9205; dilution 1:400); mouse anti-βIII-tubulin-FITC-conjugate (Abcam, ab25770; dilution 1:80); and mouse anti-Hu antigens C and D (Invitrogen, A-21271; dilution 1:300). After rinsing with 0.2% gelatin, secondary antibodies were loaded during 45 min as follows: anti-rabbit or anti-mouse Alexa488; anti-goat Alexa594; and anti-mouse Alexa647 (all Invitrogen; dilution 1:500). Nuclei were stained with Hoechst 32258 (2 μm, 10 min). A post-fixation step was performed with 2% paraformaldehyde for 10 min to prevent any dissociation of secondary antibodies, followed by a quenching step with 50 mm NH_4_Cl for 5 min. Coverslips were mounted using prolong gold antifade reagent (Invitrogen). Images of DVL2 and NRX were acquired using a TCS SP8 confocal microscope. Localization of β-catenin or neuronal markers was assessed using LSM 710 NLO confocal system.

##### FRET Measurement

To assess physical protein-protein association between DVL2 and NRX, we used FRET microscopy (22) through the sensitized emission method using a TCS SP5 confocal microscope equipped with 63× 1.4NA differential interference contrast oil HCX Plan-Apo objective. The parameter settings were kept constant throughout all experiments. The donor (Alexa488-labeling DVL2) was excited at 488 nm, and a restrictive detection wavelength range was set at 490–550 nm to avoid leakage of acceptor fluorescence into the donor image. The acceptor (Alexa594-labeling NRX) was excited at 594 nm, and the detection range was set at 620–700 nm. The FRET signal was acquired in line-by-line sequential mode. First the donor and the FRET signal were detected after the selective excitation of the donor. Then the acceptor was excited with the selective excitation light, followed by the detection of the acceptor signal only. This acquisition method involving suitable laser switching-on avoided the excitation of the other fluorochrome. We used reference samples (cell specimens labeled with donor only or acceptor only) to supply calibration coefficients for the correction of excitation and emission cross-talk (*i.e.* direct acceptor excitation and donor emission bleed-through) from the FRET specimen (cell sample labeled with both donor and acceptor). Following this procedure, the fully corrected FRET image was obtained (according to the recommendations in Ref. 23). The proportion of direct protein-protein binding, *i.e.* FRET efficiency (FRET_eff_), was calculated using the equation of Van Rheenen *et al.* (24). FRET_eff_ was measured inside individual cells (*n* = ∼100 per time point) for every condition. Measurements were performed for three independent experiments.

##### Quantitative Real Time PCR

Cell disruption and purification of total RNA were performed with high pure RNA isolation kit (Roche Applied Science) according to the manufacturer's instructions. Residual contaminating genomic DNA was digested by DNase I recombinant RNase-free (Roche Applied Science). cDNA synthesis was primed with oligo(dT)_18_ primers and generated from 1 μg of template RNA with Moloney murine leukemia virus reverse transcriptase using first strand cDNA synthesis kit (Thermo Scientific). RT-PCR was performed as follows: 5 min at 25 °C, 60 min at 37 °C, and 5 min at 70 °C. Real time PCR quantitation was performed by mixing 100 ng of template cDNA with TaqMan gene expression master mix and following TaqMan gene expression assays (all Applied Biosystems): *MCU* (Hs00293548_m1); *ITPR1* (Hs00181881_m1); *AXIN2* (Hs00610344_m1); *MAP2* (microtubule-associated protein 2; Hs00258900_m1); *RPL13A* (ribosomal protein L13a; Hs04194366_g1). Contents were transferred into 96-well PCR plates (Thermo Scientific) with a final concentration of cDNA of 5 ng/μl in each well. Amplifications were performed using iQ5 real time PCR detection system (Bio-Rad) as follows: 2 min at 50 °C for activation of the uracil-*N*-glycosylase; 10 min at 95 °C for polymerase activation; 40 repeats of two-step cycling (15 s at 95 °C for denaturation and 1 min at 60 °C for annealing and extension). Relative expression values were obtained by normalizing *C_t_* values of the tested genes in comparison with *C_t_* values of ribosomal protein L13a (*RPL13A*, housekeeping gene) using the Δ*C_t_* method (25). Each condition was assessed from three independent samples in duplicate. Results are presented as fold induction means ± S.D. from three independent experiments.

##### Statistical Analysis

Statistical analyses were performed using two-tailed unpaired Student's *t* test. *, *p* ≤ 0.05, significantly different from the control (untreated proliferating cells). Data are presented as means ± S.D. and averaged from at least three independent experiments.

## RESULTS

### 

#### 

##### Mitochondrial ROS Metabolism Is Altered during the Early Differentiation Phase of hNPCs

The onset of neural differentiation in hNPCs is regulated in a narrow time range by the tight activation of Wnt/β-catenin pathway components. Whether endogenous ROS levels could increase after GF depletion and play any role in DVL2-NRX dissociation is unclear. To assess the changes in intracellular ROS levels, we first monitored the cellular redox balance state using the redox indicator carboxy-H_2_DCFDA (16) at 10-min intervals within the 1st h, followed by 0.5-h intervals for 3 h throughout the initial phase of differentiation (Fig. 1, *B–D*). Proliferating cells showed only a faint signal (Fig. 1*B, 0h, arrows*). Upon induction of differentiation, the signal increased within 30 min (Fig. 1*B, 0.5h*), reached its maximum after 1 h, and returned to baseline after 2.5 h (Fig. 1, *B, 2.5h, arrow, C* and *D*). In parallel, we quantified the cellular redox state using flow cytometry and obtained similar results (Fig. 1*E*).

Exchange of cell culture medium can impose oxidative stress (26); however, sequential exchanges of the proliferating medium in the dye pre-loaded cells did not cause any increase in the fluorescent signal showing that neither cell stress nor redox imbalance occurs during the experimental time frame (Fig. 1*F*). The 0.5-h incubation of proliferating cells with pro-oxidant agent H_2_O_2_ (1 mm) resulted in the increased redox imbalance, as anticipated (Fig. 1, *D* and *E*). Although H_2_O_2_ cytotoxicity depends on various factors, including the dose or the incubation time (27), the cytotoxic effect of H_2_O_2_ (3 mm) was noticeable only 2 h after treatment (41%). Consistent with the reported data (28), no visible and only minor (13%) cytotoxic effects were observed after 0.5 and 1 h, respectively (Fig. 1*G*). Thus, our data attribute the increase in the intracellular redox imbalance in the first 3 h of hNPCs differentiation solely to GF depletion.

**FIGURE 2.**
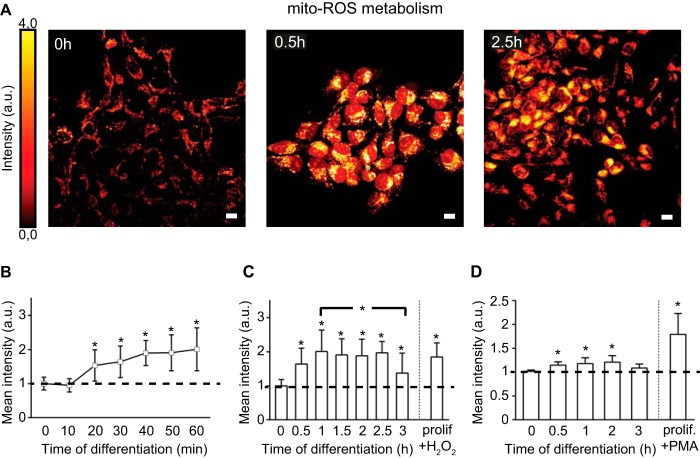
**Changes in mitochondrial ROS metabolism during differentiation.** *A,* confocal images of the mito-ROS levels (glow dark) at 0, 0.5, and 2.5 h of differentiation. *B,* kinetics of mito-ROS metabolism measured as mean fluorescent intensity at 10-min intervals over the 1st h of differentiation. *n* = ∼150 cells per time point. *C,* kinetics of mito-ROS metabolism measured as mean fluorescent intensity at 0.5-h intervals over the first 3 h of differentiation. *n* = ∼200 cells per time point. *prolif.*, proliferating. *D,* flow cytometry as parallel determination confirming the variation of ROS levels. *, *p* ≤ 0.05. *Error bars,* S.D. *Scale,* 10 μm; *a.u.*, arbitrary units.

The changes in the redox state could reflect the rise in ROS production in the mitochondria. We examined the mitochondrial ROS (mito-ROS) metabolism using the MitoTracker red marker (17). The dye accumulated in mitochondria in a quenched nonfluorescent state (Fig. 2*A, 0h*). mito-ROS production led to an increased fluorescence within 0.5 h of differentiation (Fig. 2, *A, 0.5h, B* and *C*) and dropped to baseline by 3 h (Fig. 2, *A, 2.5h,* and *C*). Using flow cytometry, we obtained similar results with cells stained with another ROS-sensitive dye, dihydrorhodamine 123 (Fig. 2*D*) (16). Treatment of proliferating cells with pro-oxidant agents, H_2_O_2_ (1 mm) or phorbol 12-myristate 13-acetate (5 μm), induced a significant increase of the fluorescent signal after 0.5 and 1 h, respectively (Fig. 2, *C* and *D*).

Our data show the dynamic changes in the redox state at the early phase of hNPC differentiation and reflect the alteration in the cellular physiology. They further reveal the existence of a switch in mitochondrial ROS production within 30 min after GF depletion.

##### GF Depletion Mediates Early ER-Ca^2+^ Release and mito-Ca^2+^ Influx

Intracellular Ca^2+^ is a ubiquitous messenger involved in various aspects of cell physiology (29), *e.g.* Ca^2+^ influx into the mitochondria stimulates ROS generation (30–32). EGF and bFGF reportedly inhibit ITPR-dependent Ca^2+^ release from intracellular stores (33, 34). We therefore asked whether GF depletion could result in changes in intracellular Ca^2+^ fluxes and affect ROS metabolism. To examine the intracellular Ca^2+^ distribution after GF depletion, we used fluorescent dyes that preferentially bind Ca^2+^ in the ER, cytosol, and mitochondria and monitored the Ca^2+^ fluxes.

**FIGURE 3.**
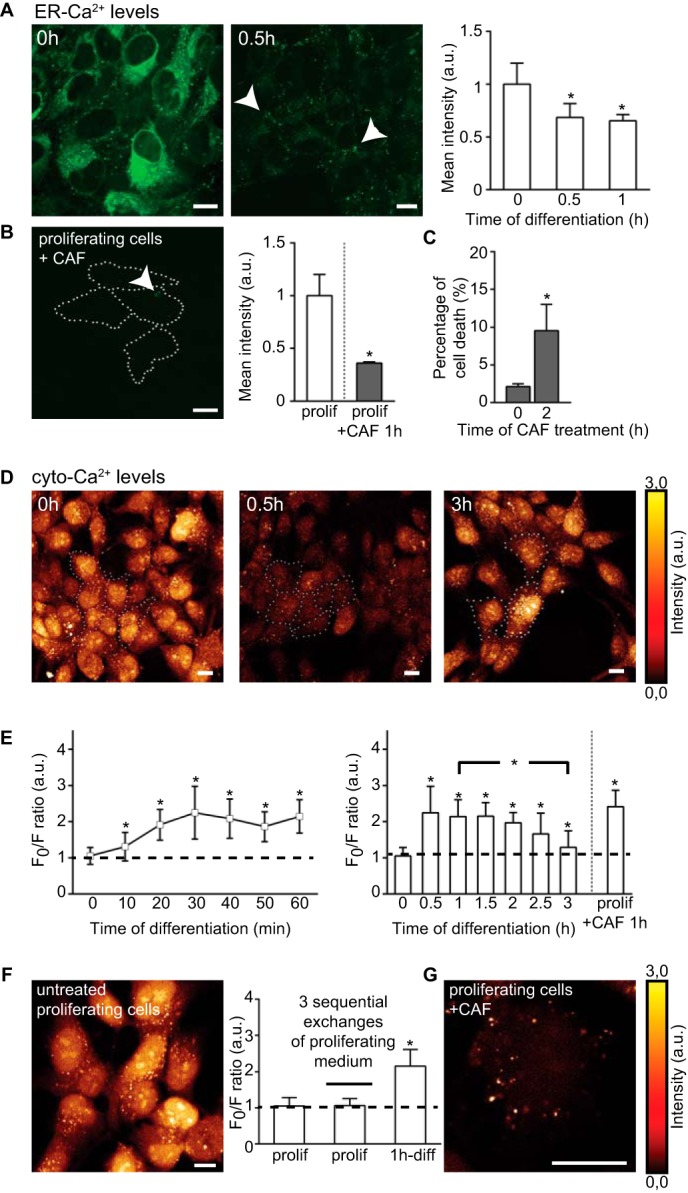
**Changes in intracellular Ca^2+^ compartmentalization during differentiation.** *A,* confocal images of ER-Ca^2+^ levels (*green*) at 0 and 0.5 h of differentiation. *White arrows* in *0.5-h panel* indicate residual ER-Ca^2+^ as punctate signal. Mean fluorescent intensities show Ca^2+^ release 30 min after initiation of differentiation. *n* = ∼100 cells per time point. *B,* confocal image of ER-Ca^2+^ levels (*green*) in proliferating (*prolif.*) cells treated for 1 h with 1 mm CAF. Cell boundaries are shown by *dotted white lines*. Only a punctate fluorescence signal remains (*white arrow*), consisting of the residual ER-stored Ca^2+^. *Histogram* shows mean fluorescent intensity values of ER-Ca^2+^. *n* = ∼50 cells per time point. *C,* cytotoxic assay of CAF treatment (10 mm) using trypan blue exclusion assay. *D,* confocal images of the Ca^2+^-unbound form of fura red (glow dark) at 0, 0.5, and 3 h of differentiation. The decrease in the signal (0.5 h) reflects the increase of the cyto-Ca^2+^ levels. *E,* kinetics of the cyto-Ca^2+^ levels. An increase at 0.5 h is followed by a return to baseline after 3 h of differentiation. *n* = ∼150 cells per time point. *F,* confocal image of cyto-Ca^2+^ levels (glow dark) after three sequential exchanges of culture medium in proliferating cells. The signal was quantified and compared with control and differentiating cells. *n* = ∼50 per time point. *G,* confocal image of Ca^2+^-unbound form of fura red (glow dark) in CAF-treated proliferating cells (10 mm, 1 h). *, *p* ≤ 0.05. *Error bars*, S.D. *Scale,* 10 μm; *a.u.*, arbitrary units.

The ER-Ca^2+^ can be detected using mag-fluo-4, which exhibits fluorescent intensity proportional to ER-Ca^2+^ levels (18). The mag-fluo-4 clearly labeled the ER network in proliferating cells (Fig. 3*A, 0h*); after 0.5 h of differentiation, the signal significantly decreased indicating the Ca^2+^ release from the ER stores (Fig. 3*A, 0.5h, arrows*). Similarly, a 1-h treatment of proliferating cells with 1 mm CAF, which stimulates Ca^2+^ release from the ER stores via the ryanodine receptors (35, 36), strongly decreased the signal (Fig. 3*B*). Of note, in agreement with reports showing CAF cytotoxicity only after longer exposures (37), a 2-h CAF treatment of proliferating cells leads to only 10% of cell death (Fig. 3*C*).

Next, we assessed the changes in cytosolic Ca^2+^ (cyto-Ca^2+^) levels after GF depletion using ratiometric fura red. The dye fluoresces in its Ca^2+^-unbound form, so an increase in cyto-Ca^2+^ concentration diminishes the signal (20, 21). Consistent with the observed ER-Ca^2+^ release, the cyto-Ca^2+^ levels in differentiating cells increased within 10 min and reached maximum by 0.5 h as detected by low levels of the Ca^2+^-unbound form (Fig. 3, *D, 0.5h,* and *E*) and returned to baseline at 3 h of differentiation (Fig. 3, *D, 3h,* and *E*). These data corroborate the inverse relationship between ER and cytosolic Ca^2+^ levels. Although intracellular Ca^2+^ levels remained unchanged in proliferating cells after the sequential exchanges of proliferative medium (Fig. 3*F*), 1 h of CAF treatment led to an increase in cyto-Ca^2+^ levels, comparable with the increase at 0.5 h in differentiating cells (Fig. 3, *E* and *G*).

**FIGURE 4.**
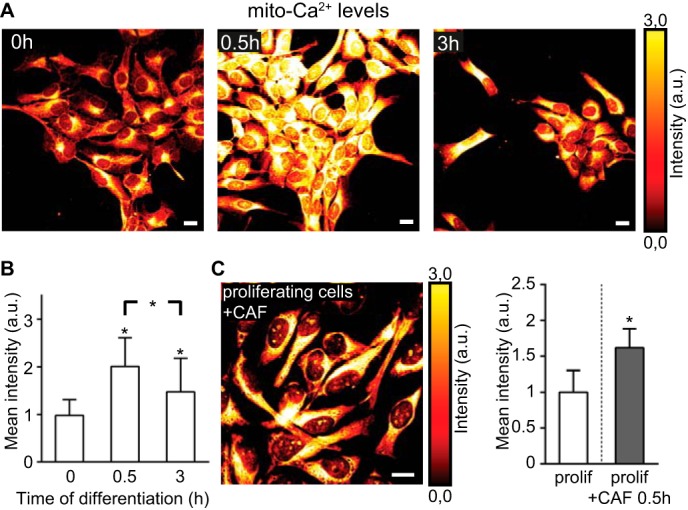
**Changes in mitochondrial Ca^2+^ during differentiation.** *A,* confocal images of mito-Ca^2+^ levels (glow dark) at 0, 0.5, and 3 h of differentiation. *B, histogram* shows mean fluorescent intensity values of mito-Ca^2+^ levels during the first 3 h of differentiation. An increase in Ca^2+^ influx into mitochondria occurs at 0.5 h of differentiation. *n* = ∼150 cells per time point. *C,* confocal image of mito-Ca^2+^ levels (glow dark) in CAF-treated proliferating (*prolif.*) cells (1 mm, 0.5 h). *Histogram* shows mean fluorescent intensity values of mito-Ca^2+^. *n* = ∼100 cells per time point. *, *p* ≤ 0.05. *Error bars*, S.D. *Scale,* 10 μm; *a.u.*, arbitrary units.

To assess the mito-Ca^2+^ levels, we used x-rhod-1, a dye that preferentially accumulates in mitochondria and fluoresces proportionally to the Ca^2+^ levels in these organelles (19). The mito-Ca^2+^ signal markedly rose after 0.5 h of differentiation indicating an increased mitochondrial Ca^2+^ influx (Fig. 4, *A, 0h, 0.5h,* and *B*), and decreased by 3 h of differentiation (Fig. 4, *A, 3h,* and *B*). CAF treatment of proliferating cells also increased mito-Ca^2+^ levels (Fig. 4*C*) corroborating the data observed in differentiating cells. Altogether, these data indicate that GF depletion leads to an increased Ca^2+^ efflux from the ER to the cytosol and a subsequent Ca^2+^ influx into the mitochondria within the first 30 min of differentiation.

##### mito-Ca^2+^ Uptake Triggers the ROS Metabolism

**FIGURE 5.**
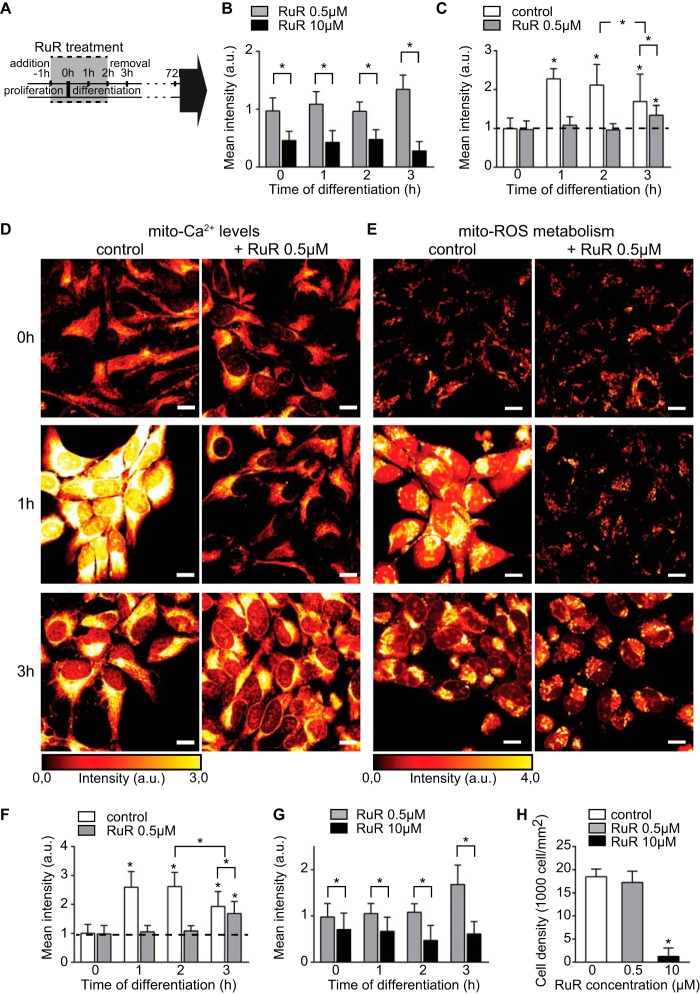
**Mitochondrial Ca^2+^ fluxes regulate ROS metabolism.** *A,* schematic of the 3-h RuR treatment as follows: 1 h treatment of proliferating cells with RuR + GF is followed by a 2-h treatment of differentiating cells with RuR-GF. The medium is replaced by a drug-free GF-free medium at 2 h of differentiation. *B, histogram* shows mean fluorescent intensity values of mito-Ca^2+^ levels for low (*gray*) and high RuR dose (*black*). *n* = ∼100 cells per time point. *C,* kinetics of mito-Ca^2+^ levels in untreated (*white*) and low RuR dose-treated cells (*gray*) plotted as mean fluorescent intensities. *n* = ∼150 cells per time point. *D,* confocal images of mito-Ca^2+^ levels (glow dark) in untreated and 0.5 μm RuR-treated cells at 0, 1, and 3 h of differentiation. 0.5 μm RuR inhibits Ca^2+^ influx within mitochondria, but its effect is reversible. *E,* confocal images of mito-ROS metabolism (glow dark) in untreated and 0.5 μm RuR-treated cells at 0, 1, and 3 h of differentiation. RuR prevents the rise of ROS production. *F,* kinetics of mito-ROS levels plotted in untreated (*white*) and low RuR dose-treated cells (*gray*) as mean fluorescent intensities. Drug removal reverses the effect shown by significant elevation of mito-ROS levels at 3 h of differentiation. *n* = ∼300 cells per time point. *G, histogram* shows mean fluorescent intensity values of mito-ROS levels for low (*gray*) and high RuR dose (*black*). *n* = ∼100 cells per time point. *H,* cells were seeded at the same concentration prior to RuR treatment, and the induction of differentiation and cell number was scored at 72 h of differentiation. *Histogram* shows mean cell density values per individual treatments and untreated cells. *, *p* ≤ 0.05. *Error bars*, S.D. *Scale,* 10 μm; *a.u.*, arbitrary units.

The observed intracellular Ca^2+^ dynamics occur concomitantly with the changes in mito-ROS metabolism and cellular redox state (compare Figs. 1, *C* and *D*, 2, *B* and *C*, 3*E,* and 4*B*). RuR reportedly blocks the mito-Ca^2+^ uptake by inhibiting the MCU, the main conduit for mitochondrial Ca^2+^ influx (38, 39). We prevented mito-Ca^2+^ influx by incubating cells with RuR (see under “Experimental Procedures” for details, Fig. 5*A*), and we monitored Ca^2+^ levels within mitochondria for 3 h. The effect of RuR was dose-dependent; at 0 h, the high dose (10 μm) strongly reduced the signal indicating the decrease of baseline mito-Ca^2+^ levels, whereas the low dose (0.5 μm) did not cause any significant change in comparison with untreated cells (Fig. 5, *B–D, 0h*). At 1 h, Ca^2+^ accumulated within the mitochondria in untreated cells, whereas 0.5 μm RuR prevented mito-Ca^2+^ uptake (Fig. 5, *C* and *D, 1h*). At 3 h, the mito-Ca^2+^ levels dropped to baseline in the untreated cells. Conversely, after 0.5 μm RuR removal, mito-Ca^2+^ levels significantly increased indicating that the drug effect is reversible (Fig. 5, *C* and *D*, *3h*). The effect of the high dose was long lasting, and the mito-Ca^2+^ levels were decreased for the entire examined period (Fig. 5*B*).

To assess the functional link between mito-Ca^2+^ and ROS metabolism at the onset of hNPC differentiation, we examined the mito-ROS production after inhibiting Ca^2+^ influx into the mitochondria. At 0 h, mito-ROS levels in 0.5 μm RuR-treated cells were comparable with the untreated cells (Fig. 5, *E, 0h,* and *F*). After 1 h of differentiation, the low dose prevented the rise in ROS levels (Fig. 5, *E, 1h,* and *F*). However, 1 h after the drug removal, its effect was reversed, and the mito-ROS production was elevated (Fig. 5, *E, 3h,* and *F*). The high dose drastically reduced the signal and completely abolished mitochondrial metabolism, presumably due to its cytotoxicity (Fig. 5, *G* and *H*). The low dose did not cause any significant cell death (Fig. 5*H*).

These data demonstrate that the effect of short term treatment with 0.5 μm RuR is nonlethal and reversible, and inhibits both mito-Ca^2+^ uptake and ROS increase. Moreover, our findings suggest that mito-ROS metabolism is triggered by a rise in mito-Ca^2+^ levels at the onset of differentiation.

##### Ca^2+^-mediated ROS Metabolism Dissociates the DVL2-NRX Complex

**FIGURE 6.**
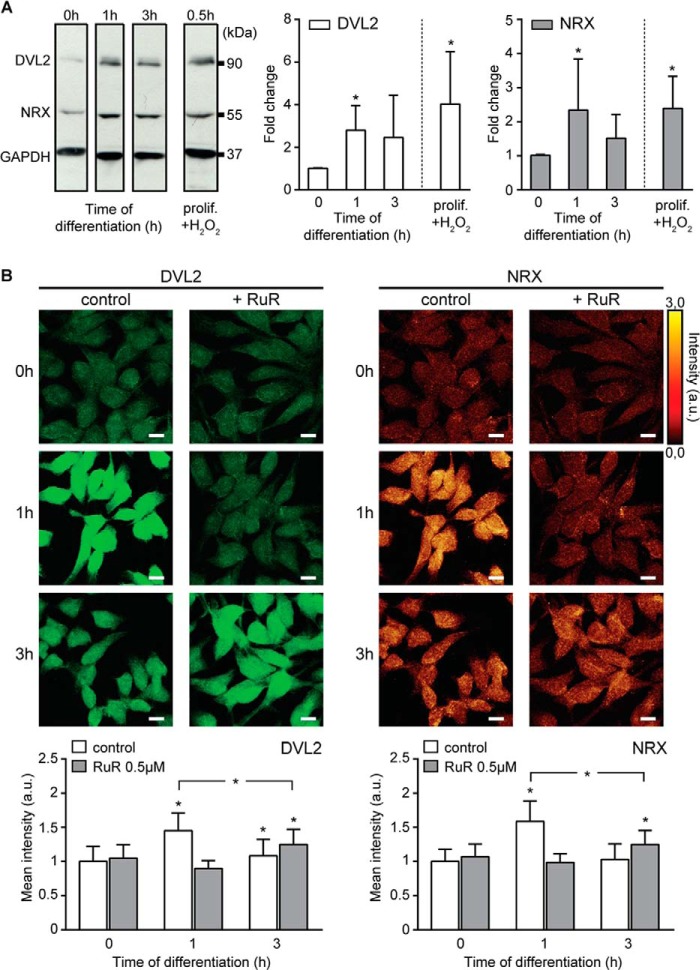
**DVL2 and NRX amounts are affected by Ca^2+^-mediated ROS metabolism.** *A,* Western blots of DVL2 and NRX showing a ROS-dependent increase of both protein amounts occurring 1 h after differentiation or after H_2_O_2_ treatment (1 mm). All bands are from the same blot. The signal intensities were normalized to 1 at 0 h (proliferating cells) and quantified as a fold change. *B,* confocal images of DVL2 (*green*) and NRX (glow dark) in untreated and 0.5 μm RuR-treated cells at 0, 1, and 3 h of differentiation. RuR inhibits the increase of both proteins (1 h) and removal of RuR allows for the cytosolic accumulation of both proteins (3 h). Mean fluorescent intensities are quantified in *bar graphs. n* = ∼200 cells per time point. *, *p* ≤ 0.05. *Error bars*, S.D. *Scale,* 10 μm; *a.u.*, arbitrary units.

The GF depletion results in the activation of Wnt/β-catenin pathway that is required for induction of hNPC differentiation (14, 15, 40). One way to modulate the Wnt/β-catenin pathway activity is to regulate the availability of DVL2. The exogenously triggered ROS production leads to the dissociation of DVL from its complex with NRX (9). We therefore hypothesized that the endogenous changes in ROS levels mediated by mito-Ca^2+^ fluxes can tune the activation of the Wnt/β-catenin cascade in hNPCs. First, we verified the reported data showing the early kinetics of DVL2 activity in these cells (15). Through Western blotting and immunocytochemistry, we confirmed that DVL2 protein levels are elevated within 1 h after GF depletion, followed by the moderate reduction at 3 h (Fig. 6, *A* and *B*). Markedly, we found that NRX protein levels followed the same pattern; the NRX accumulated within 1 h, and its levels decreased by 3 h (Fig. 6, *A* and *B*). If the DVL2 and NRX accumulation were mediated through the increase in intracellular ROS, the treatment of proliferating cells with pro-oxidant agent would result in higher DVL2 and NRX protein levels. Conversely, inhibiting the ROS production would prevent the DVL2 and NRX increase. Indeed, although DVL2 and NRX proteins were significantly elevated after 1 mm H_2_O_2_ treatment of proliferating cells (Fig. 6*A*), the incubation of differentiating cells with 0.5 μm RuR delayed the accumulation of both DVL2 and NRX proteins (Fig. 6*B, control 1h versus RuR 3h*). These data suggest that preventing mitochondrial ROS formation affects cytoplasmic DVL2 as well as NRX protein levels.

**FIGURE 7.**
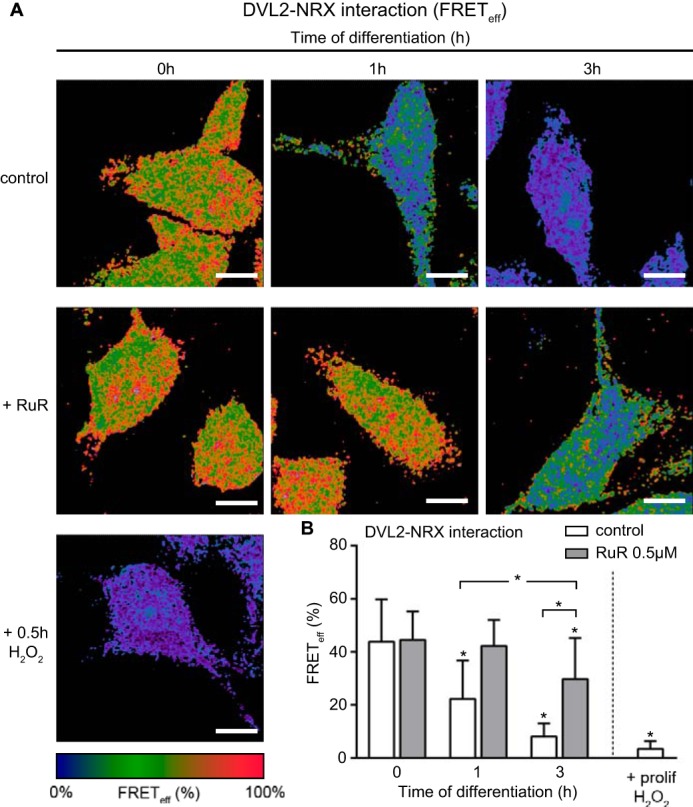
**Ca^2+^-mediated ROS metabolism modulates DVL2-NRX complex.** *A,* representative pseudocolor images illustrating FRET_eff_ resulting from the fully corrected FRET signal at 0, 1, and 3 h of differentiation in untreated and 0.5 μm RuR-treated cells and in 1 mm H_2_O_2_-treated proliferating cells. The *color scale* at the bottom of the panel is shown in %. *B,* mean values of FRET_eff_ of images shown in *A*. FRET_eff_ in untreated cells is reduced by half in 1 h after GF depletion, although it remains unchanged in RuR-treated cells. 1 h after RuR removal, DVL2-NRX complex begins to dissociate as shown by ∼30% reduction in FRET_eff_, *n* = ∼100 cells per time point. *prolif.,* proliferating. *, *p* ≤ 0.05. *Error bars,* S.D. *Scale,* 10 μm.

To address whether the altered redox and metabolic cell states could mediate the dissociation of DVL2 from NRX, we examined the physical association between the two proteins by fluorescence resonance energy transfer (FRET) microscopy for a period of 3 h after GF removal (23, 24). At 1 h after differentiation, the proportion of DVL2 bound to NRX as measured by FRET efficiency(FRET_eff_) significantly decreased (Fig. 7, *A, control, 1h,* and *B, empty histograms*). The H_2_O_2_ treatment of proliferating cells also resulted in decreased FRET_eff_ (Fig. 7, *A, H*_2_*O*_2_, and *B*). Markedly, 0.5 μm RuR treatment inhibited the dissociation of DVL2-NRX complexes. FRET_eff_ between DVL2 and NRX was high in RuR-treated cells 1 h after differentiation (Fig. 7, *A, RuR, 1h,* and *B, full histograms*). Drug removal promoted dissociation of DVL2 from NRX as the FRET_eff_ decreased (Fig. 7, *A, RuR, 3h,* and *B*). These data show that the mito-Ca^2+^-mediated physiologic increase in ROS levels may facilitate fast dissociation rate of DVL2 from the initial pool of DVL2-NRX complexes. DVL2 and NRX proteins accumulate in the cytoplasm as a result of their reduced physical interactions. In the absence of elevated ROS production, DVL2 is kept inactive in the cytoplasm by binding to NRX.

##### Nuclear Accumulation of β-Catenin Is Enhanced by Increase in Ca^2+^-mediated ROS Metabolism

**FIGURE 8.**
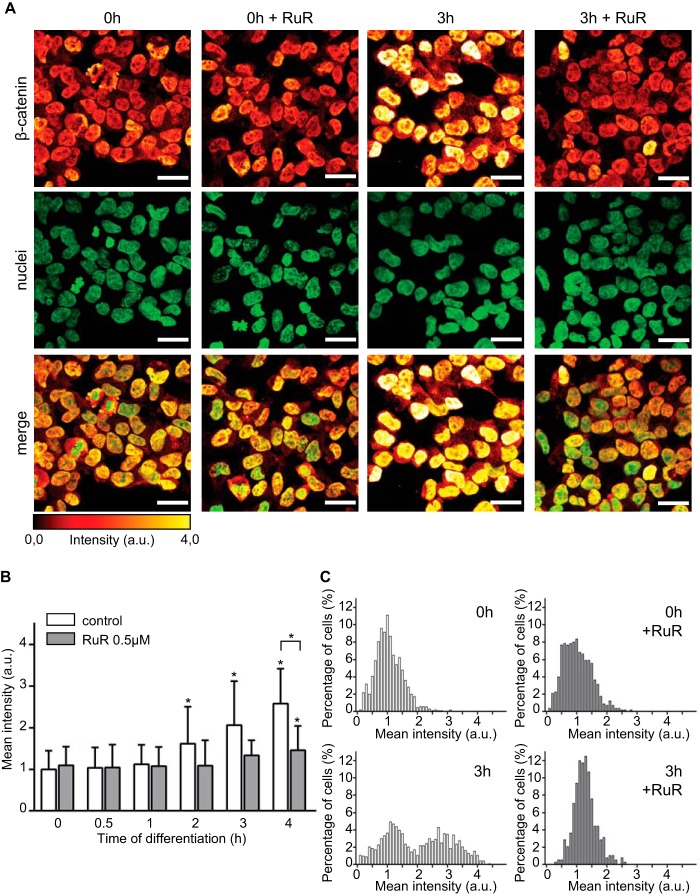
**Nuclear accumulation of β-catenin is regulated by Ca^2+^-mediated ROS metabolism.** *A,* confocal images of β-catenin (glow dark) showing that RuR treatment inhibits its nuclear accumulation. Nuclei are shown in *green*. Mitotic cells are shown by *asterisks. a.u.*, arbitrary units. *B,* mean intensities of nuclear β-catenin; *C, histograms* of frequency distribution of the fluorescent intensities show increased nuclear accumulation of β-catenin and formation of two cell populations, with and without nuclear β-catenin. RuR treatment prevents the formation of the population with nuclear β-catenin. *n* = ∼600 cells per time point. *, *p* ≤ 0.05. *Error bars*, S.D. *Scale,* 20 μm.

As a result of Wnt/β-catenin pathway activation, β-catenin accumulates in the cytoplasm and shuttles to the nucleus (41). In the nucleus, β-catenin binds TCF/LEF1 transcription factors and drives the expression of specific target genes that control cellular processes ranging from proliferation to cell differentiation (41–43). We reasoned that blocking Ca^2+^-mediated ROS metabolism would decrease the pool of unbound DVL2 and inactivate the transduction cascade. We therefore examined the ability of β-catenin to localize into the nucleus in RuR-treated cells. At 0 h, the nuclear β-catenin signal was identical between untreated and RuR-treated cells (Fig. 8*A, 0h versus 0h*+*RuR*). After 3 h of differentiation, β-catenin accumulated in the nuclei of untreated cells (Fig. 8*A, 3h*) confirming previous reports (15). In contrast, RuR treatment prevented nuclear accumulation of β-catenin in differentiating hNPCs (Fig. 8*A, 3h*+*RuR*). We monitored the kinetics of β-catenin nuclear localization by quantifying its mean fluorescence intensities along the first 4 h of differentiation (Fig. 8*B*). The nuclear β-catenin signal was significantly enhanced in untreated cells from 2 h of differentiation onward (Fig. 8*B, empty histograms*). Frequency distribution analysis revealed that at 3 h of differentiation, two cell populations emerge as follows: the first one, in which β-catenin levels are comparable with proliferating cells, and the second one with twice as high mean intensities representing the cells with the accumulated nuclear β-catenin (Fig. 8*C, 3h versus 0h*). RuR treatment prevented the two cell populations to emerge (Fig. 8*C, 0h*+*RuR versus 3h*+*RuR*), but a moderate increase in the nuclear mean intensities of β-catenin occurred after drug removal (Fig. 8*B, full histograms, 4h*). These data indicate that Ca^2+^-mediated ROS metabolism enhances the nuclear β-catenin accumulation and may affect the rate of its nucleo-cytoplasmic shuttling.

##### Ca^2+^-mediated ROS Metabolism Augments the Wnt/β-Catenin Signaling

**FIGURE 9.**
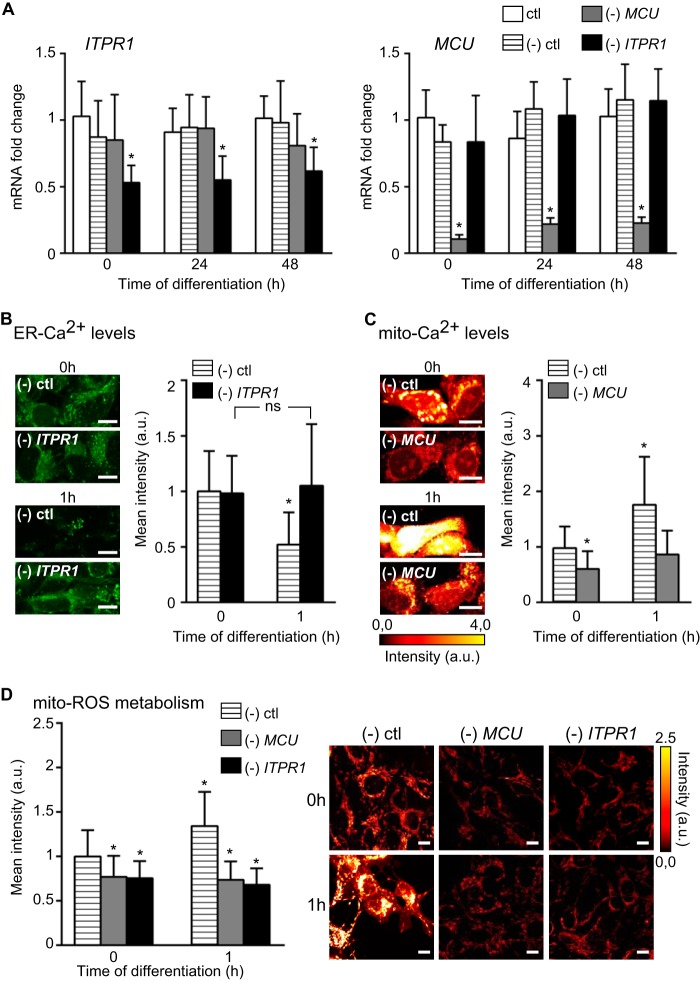
**ITRP1 and MCU activities regulate the Ca^2+^-mediated ROS metabolism.** *A, ITPR1* and *MCU* mRNA levels (fold changes) were analyzed by quantitative real time PCR and expressed at 0, 24, and 48 h after the differentiation was initiated. Data for untreated cells (*ctl*) were compared with cells transfected with *ITPR1*, *MCU*, or untargeted (−*) ctl* siRNAs. *B,* confocal images of ER-Ca^2+^ levels (*green*) in (−) control and (−) *ITPR1* at 0 and 1 h of differentiation. The *bar graphs* present the mean fluorescent intensity values (*a.u.*). *a.u.*, arbitrary units. *ITPR1* silencing leads to the sequestration of Ca^2+^ in the ER, although Ca^2+^ is released in (−) control. *n* = ∼100 cells per time point. *C,* confocal images of mito-Ca^2+^ levels (glow dark) in (−) control and (−) *MCU* at 0 and 1 h of differentiation. The *bar graphs* show the mean fluorescent intensity values. *MCU* silencing prevents Ca^2+^ accumulation in mitochondria. *n* = ∼100 cells per time point. *D,* confocal images of mito-ROS levels (glow dark) in (−) control, (−) *MCU*, and (−) *ITPR1* at 0 and 1 h of differentiation. The *histograms* present the mean fluorescent intensity values. Both *MCU* and *ITPR1* silencing block the rise of ROS metabolism. *n* = ∼100 cells per time point. *, *p* ≤ 0.05. *Error bars*, S.D. *Scale,* 5 μm.

To definitively demonstrate that Ca^2+^-mediated ROS metabolism modulates the Wnt/β-catenin signaling, we genetically perturbed the ER and mitochondrial Ca^2+^ fluxes and examined their effects on Wnt target gene expression during hNPC differentiation. We reduced *ITPR1* (mammalian brain predominant isoform (44)) to inhibit Ca^2+^ release from the ER stores, and *MCU* to block the Ca^2+^ mitochondrial influx (39). We first assessed the silencing efficiency for both *ITPR1* and *MCU*; *ITPR1* expression was stably reduced by 50% and MCU by 90% as compared with untransfected cells and cells transfected with untargeted siRNA (control cells) (Fig. 9*A*). Next, we examined the functional effects of *ITPR1* and MCU silencing. At 0 h, the ER-Ca^2+^ levels were comparable between control cells and cells with the reduced *ITPR1* (Fig. 9*B*). At 1 h of differentiation, ER is depleted of Ca^2+^ by 50% in control cells (compare with Fig. 3*A*), in contrast loss of *ITPR1* completely blocks ER-Ca^2+^ release after GF depletion (Fig. 9*B*). Similarly, Ca^2+^ influx into mitochondria is increased after 1 h of differentiation in control cells (by ∼80%) (compare with Fig. 9*C* to Figs. 4, *A* and *B*, and 5, *C* and *D*) but is significantly suppressed in the absence of *MCU*. Of note, the basal levels of mito-Ca^2+^ are mildly affected in proliferating cells transfected with *MCU* siRNA. These changes in Ca^2+^ fluxes have a profound effect on ROS metabolism. Although in control cells ROS levels significantly increase 1 h after differentiation (see also Figs. 2, *A–C,* and 5, *E* and *F*), loss of both *ITPR1* and *MCU* affect the ROS metabolism already in proliferating cells and diminish the mito-ROS production after induction of differentiation (Fig. 9*D*), indicating that Ca^2+^ flux through MCU is critical for cellular bioenergetics as suggested previously (45). The data show that Ca^2+^ influx into mitochondria triggers mito-ROS metabolism, corroborating the specificity of RuR.

**FIGURE 10.**
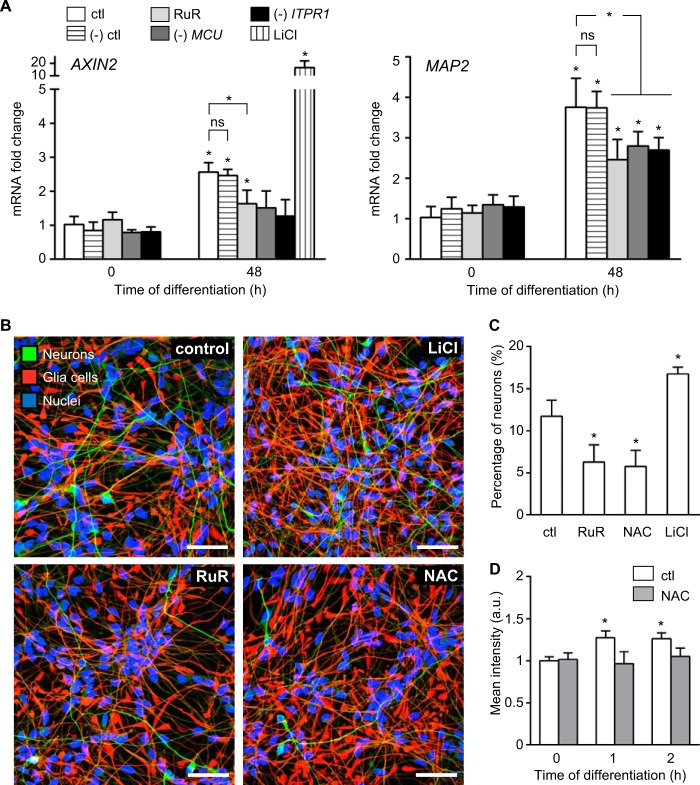
**Ca^2+^-mediated ROS metabolism modulates β-catenin-dependent neuronal differentiation.** *A, AXIN2* and *MAP2* mRNA levels detected by quantitative real time PCR at 0 and 48 h after the differentiation was induced. Data for untreated cells (*ctl*) are compared with cells treated with RuR or LiCl, and with transfected cells as follows: (−) control, (−) *MCU*, and (−) *ITPR1*. Results show that both chemical and genetic disruption of Ca^2+^-mediated ROS metabolism down-regulated the gene response of *AXIN2* and *MAP2. B,* confocal images of neurons (*green*) and glia cells (*red*) 3 days after initiation of differentiation showing less neurons when cells were treated with 0.5 μm RuR or 10 mm NAC, although 20 mm LiCl increased the neuronal amount. Nuclei are shown in *blue. C, bar graph* shows the quantification of the neuronal yields at 3 days of differentiation. *n* = ∼5000 cells per condition. *D,* intracellular ROS levels measured in untreated and NAC-treated cells using flow cytometry. Fluorescence intensities of dihydrorhodamine 123 (DHR123) were averaged at 0, 1, and 2 h after induction of differentiation. *, *p* ≤ 0.05. *Error bars*, S.D. *Scale,* 50 μm; *a.u.*, arbitrary units. *ns,* not significant.

We then addressed whether Wnt/β-catenin signaling is affected when Ca^2+^-mediated ROS metabolism is perturbed. We tested the expression levels of two Wnt/β-catenin pathway target genes: *AXIN2* and *MAP2*, a neuron-specific microtubule-associated protein 2 (46, 47). We observed 3-fold increase in *AXIN2* and 4-fold increase in *MAP2* expression 2 days after differentiation in untreated and control cells (Fig. 10*A*); a similar increase in Wnt target gene expression was reported previously (15). In cells treated with 20 mm lithium chloride (LiCl, a potent Wnt pathway agonist (48)), *AXIN2* levels increased by 20-fold (Fig. 10*A*). In contrast, RuR treatment as well as loss of *ITPR1* and *MCU* significantly reduced the *AXIN2* and *MAP2* mRNA levels at this time point (Fig. 10*A*). Taken together, our findings demonstrate that Ca^2+^ release from the ER at the onset of hNPC differentiation depends, at least in part, on ITPR1 activity. Subsequent Ca^2+^ influx into the mitochondria requires MCU and stimulates ROS production. Moreover, these data show that reduced Ca^2+^-mediated mitochondrial ROS metabolism has a negative impact on Wnt target gene expression.

##### Ca^2+^-mediated ROS Metabolism Potentiates the Wnt/β-Catenin-dependent Neuronal Differentiation

Our findings that Ca^2+^-mediated ROS metabolism enhances the Wnt/β-catenin signaling output infer that the neuronal yield of differentiating hNPCs could be modulated by the mitochondrial Ca^2+^ influx and ROS production. To test the effect of Ca^2+^-mediated ROS metabolism perturbations on hNPC neural fate commitment, we quantified the neuronal yield after 3 days of differentiation in cells with disrupted ROS metabolism. We treated the cells either with 0.5 μm RuR or 10 mm NAC, a potent ROS scavenger that protects against oxidative stress (49). Upon GF removal, 12% of the untreated cells differentiated into neurons (Fig. 10, *B* and *C*). Inhibiting mito-Ca^2+^ uptake with RuR had a profound effect on the neuronal yield. Only 6% of the cells differentiated into neurons (Fig. 10, *B* and *C*). Similarly, NAC also reduced the neuronal yield to 6% (Fig. 10, *B* and *C*). NAC treatment efficiently blocked the ROS increase at the onset of hNPC differentiation, indicating its high antioxidant capacity (Fig. 10*D*). Conversely, stimulating Wnt/β-catenin signaling with LiCl significantly enhanced the neuronal yield up to 17%, confirming that the induction of the Wnt/β-catenin pathway promotes the neuronal differentiation in our cell model. Of note, the proportion of differentiated neurons of the RuR-treated cells at 3 days was comparable with that observed with untreated cells at 2 days after GF depletion indicating delayed and ineffective differentiation (Figs. 1*A versus* 10*C*). Altogether, we demonstrate that a change in Ca^2+^-mediated mitochondrial ROS metabolism at the onset of hNPC differentiation is required for fine-tuning the Wnt/β-catenin signaling efficiency, which in turn controls the decision of the cells to commit to their neuronal fate.

## DISCUSSION

In our study, we provide direct evidence that an endogenous increase in mitochondrial ROS metabolism accelerates the rate of DVL2 dissociation from its complex with NRX and augments Wnt/β-catenin signaling efficiency. We further unravel the mechanisms that regulate ROS production in the initial phase of neural differentiation in hNPCs. At steady state, GF suppresses ER-Ca^2+^ release through ITPR1. Upon GF removal, the inhibition is alleviated and Ca^2+^ floods the cytoplasm. The increased intracellular Ca^2+^ concentration drives a number of responses, one of which involves the opening of MCU at the outer mitochondrial membrane. The increased mitochondrial Ca^2+^ influx changes the oxidative state of the mitochondria and triggers the endogenous ROS production. Importantly, loss of *ITPR1* or *MCU* abolished Ca^2+^ influx into the mitochondria and subsequent increase in ROS metabolism.

ROS involvement in stimulation of the Wnt/β-catenin pathway has been previously described in a number of cellular processes, including cell proliferation or regeneration (5–8). An increase in ROS levels is prerequisite for preventing binding of DVL2 to NRX, but the sources of ROS production remain incompletely understood. The changes in ROS levels could be stimulated by the use of exogenous pro-oxidant compound such as H_2_O_2_, by exogenous insult such as surgical amputation, or by induction of expression of NADPH oxidases (5–10). Here, we demonstrate that physiologically induced mitochondrial ROS production is necessary to dissociate DVL2-NRX complexes in a time-dependent manner. Our data further show that both DVL2 and NRX proteins accumulate in the cytoplasm and cease to interact despite their elevated protein levels. Further studies need to clarify what leads to DVL2 and NRX cytosolic accumulation or how much of the total DVL pool is in fact controlled by its interaction with NRX.

The cytosolic β-catenin accumulation and translocation into the nucleus are essential to the activation of the Wnt/β-catenin pathway (41–43). GF withdrawal increased the β-catenin nuclear accumulation from 2 h after induction of differentiation (this study and Ref. 15). We find that β-catenin nucleo-cytoplasmic shuttling occurs only after the rise in Ca^2+^-mediated ROS levels and after DVL2-NRX complexes are dissociated. Remarkably, blocking the Ca^2+^-mediated ROS metabolism either pharmacologically or genetically suppressed nuclear β-catenin accumulation as well as Wnt/β-catenin gene target activation. Moreover, interfering with the Ca^2+^-mediated ROS metabolism at the onset of differentiation suppressed the long term neuronal differentiation of hNPCs. Our findings demonstrate that Ca^2+^-mediated ROS metabolism modulates the neural differentiation and requires the release of high levels of free DVL2. We propose that such an en masse release of DVL2 ensures a robust and more efficient activation of Wnt/β-catenin signaling.

**FIGURE 11.**
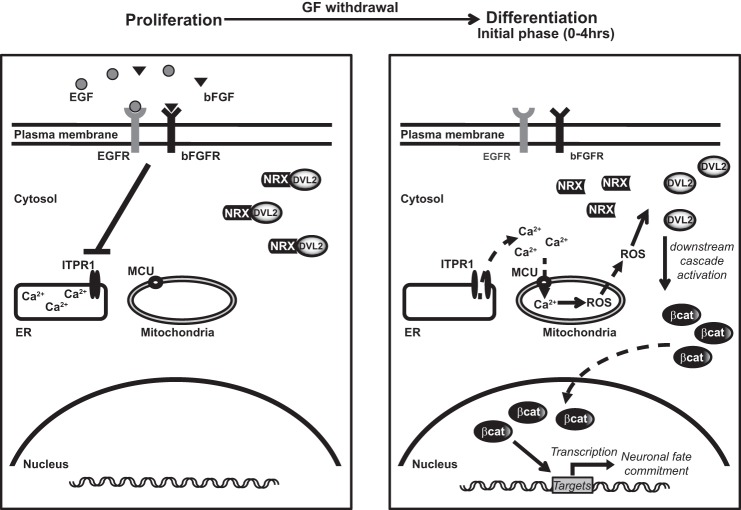
**Schematic model of neuronal differentiation of human neural progenitors mediated by Ca^2+^-dependent mitochondrial ROS metabolism.** *Dashed arrows,* fluxes; *full arrows,* stimulation; β-*cat,* β-catenin.

The following model summarizes the spatio-temporal dynamics of the successive events (Fig. 11): while the stimulation of EGF and bFGF retains Ca^2+^ in the ER stores during the proliferation phase (Fig. 11, *left panel*), their withdrawal leads to ITPR1-dependent Ca^2+^ release from the ER to the cytosol and initiates neural differentiation (Fig. 11, *right panel*). Ca^2+^ then accumulates in mitochondria through MCU resulting in the change in the electron transport chain activity, which significantly enhances ROS metabolism. ROS interacts with redox-sensitive targets such as NRX. In the proliferating cells, NRX binds DVL2 and keeps it inactive in the cytoplasm. Upon ROS elevation, NRX releases DVL2, which becomes available to further activate the downstream signaling cascade. As a consequence, cytosolic β-catenin accumulates and shuttles to the nucleus to drive specific expression of target genes involved in neuronal differentiation. At the steady state, mitochondrial Ca^2+^ returns to baseline level and the stimulation of mitochondrial ROS production is alleviated.

The metabolic switch induced by GF removal provides the cells with a cue to change from their proliferative to the differentiated state. Although Wnt/β-catenin signaling induces differentiation through regulation of target gene expression, we show that the increased ROS metabolism is required for modulating the extent of the initial signaling response. The advantage of our model system is that we can directly determine the Wnt/β-catenin signaling efficiency at the different steps of the transduction cascade. When we inhibited the Ca^2+^-mediated ROS metabolism, we observed not only a delayed dissociation of DVL2 from NRX but also a slower rate in β-catenin nuclear accumulation and lowered Wnt/β-catenin target gene expression. Consequently, the neural differentiation is attenuated as quantified by the reduced neuronal yield. Releasing the block on ROS production led to partial recovery in the protein levels of Wnt/β-catenin pathway effectors. Already 1 h after the exchange with drug-free medium, we showed a significant increase in both mito-Ca^2+^ and ROS levels with simultaneous rise in DVL2 and NRX cytoplasmic protein levels. In addition, we also detected elevated nuclear β-catenin 2 h after the drug depletion suggesting a partial rescue. These data indicate that the response amplitudes of Wnt/β-catenin pathway effectors could be modulated by physiologic changes in ROS metabolism.

Our data imply ROS in modifying the cellular decisions, in our case the neural commitment of hNPCs and their differentiation into neurons. Furthermore, the findings also demonstrate that the timing of the elevated ROS production is crucial. ROS are able to tune the intensity and the magnitude of the Wnt/β-catenin pathway responses right at the onset of neural differentiation. In conclusion, this study shows for the first time that endogenously induced changes in mitochondrial ROS metabolism positively regulate Wnt/β-catenin signal transduction and demonstrates that endogenous ROS act as permissive signals in modulating the extent of the Wnt/β-catenin signaling output. Wnt/β-catenin signaling is not the only developmental pathway regulating the neural commitment of hNPCs (50). Given the function of ROS as second messengers in many different aspects of cell physiology, the question whether ROS could modulate the neural differentiation of hNPCs through other pathways remains to be determined.

The ability of hNPCs to promptly differentiate into neurons within 3 days upon GF removal significantly reduces not only the time range for *in vitro* studies of molecular and cellular mechanisms regulating neurogenesis but also encourages the exploitation of these cells in studies directed toward stem cell therapies. Our findings imply that modulating intracellular Ca^2+^ fluxes and/or ROS metabolism in hNPCs prior to their neuronal fate commitment phase facilitates their neurogenesis. This could be particularly beneficial for enhancing *in vitro* neuronal yields and for using hNPCs as a potential neuronal source for cell replacement and regenerative therapies in the treatment of neurodegenerative diseases. Conversely, our finding that a potent ROS scavenger NAC negatively regulates neuronal differentiation prompts the re-evaluation of the therapeutic administration of antioxidants, at least when applied in the context of neuronal development as suggested previously (3).

Ca^2+^ has been shown to interact with the Wnt signaling pathway at many different levels; its role as a second messenger downstream of DVL-mediated Wnt signaling is well established (51). Recently, it has been shown that Wnt signaling mediates extracellular Ca^2+^ fluxes through regulation of L-type Ca^2+^ channel conductance (52). In this study, we show that Ca^2+^-dependent events are essential for robust activation of DVL and positively regulate Wnt/β-catenin signal transduction ensuring more efficient and sustained signaling. The data infer that ionic and metabolic cues are an integral part of the Wnt/β-catenin signaling pathway and may fine-tune the temporal element of signal transduction as well as magnitudes of responses of the pathway components.

## Footnotes

2 The abbreviations used are:

ROS: reactive oxygen species
bFGF: basic fibroblast growth factor
CAF: caffeine
carboxy-H_2_DCFDA: 6-carboxy-2′,7′-dichlorodihydrofluorescein diacetate
ER: endoplasmic reticulum
FRET_eff_: fluorescence resonance energy transfer efficiency
GF: growth factor
H_2_O_2_: hydrogen peroxide
hNPC: human neural progenitor cell
ITPR1: inositol 1,4,5-triphosphate receptor, type 1
MCU: mitochondrial calcium uniporter
NAC: *N*-acetyl-l-cysteine
NRX: nucleoredoxin
RuR: ruthenium red
mito-Ca^2+^: mitochondrial Ca^2+^
MTT: 3-(4,5-dimethylthiazol-2-yl)-2,5-diphenyltetrazolium bromide.
